# 105. Impact of a Multiplex Polymerase Chain Reaction Panel on Duration of Empiric Antibiotic Therapy in Suspected Bacterial Meningitis

**DOI:** 10.1093/ofid/ofab466.105

**Published:** 2021-12-04

**Authors:** Justin J Choi, Lars Westblade, Lee S Gottesdiener, Kyle Liang, Han A Li, Graham T Wehmeyer, Marshall J Glesby, Matthew Simon

**Affiliations:** 1 Weill Cornell Medicine of Cornell University, New York, NY; 2 Weill Cornell Medicine, New York, NY; 3 New York-Presbyterian Hospital, New York, New York

## Abstract

**Background:**

Multiplex polymerase chain reaction (PCR) panels allow for rapid detection or exclusion of pathogens causing community-acquired meningitis and encephalitis (ME). However, the clinical impact of rapid multiplex PCR ME panel results on the duration of empiric antibiotic therapy is not well characterized.

**Methods:**

We performed a retrospective pre-post study to evaluate the implementation of the FilmArray ME panel (BioFire Diagnostics, LLC) for diagnosis of bacterial meningitis at our institution. We included adults who presented with suspected bacterial meningitis, received empiric antibiotic therapy, and underwent cerebrospinal fluid microbiological testing in the emergency department. The primary outcome was duration of empiric antibiotic therapy. A bivariable analysis that compared baseline demographics, clinical characteristics, and study outcomes between the pre-ME panel and post-ME panel periods was performed using Mann-Whitney tests, chi-squared tests, or Fisher’s exact tests. Time-to-event analysis used the Kaplan-Meier method and log-rank statistics.

**Results:**

In the pre-ME panel period, the positive detection rate of bacterial pathogens was 2.2% (3/137) by cerebrospinal fluid culture and 4.3% (3/69) in the post-ME panel period. Table 1 shows baseline characteristics of patients. Compared to the pre-ME panel period, there were significant reductions in the post-ME panel period for the duration of empiric antibiotic therapy (median 34.7 h, IQR 8.5–61.7, vs. 12.3 h, IQR 3.3–40.0, *P*=0.01), time to targeted therapy (59.3 h, IQR 36.5–74.6, vs 7.02 h, IQR 0.9–12.4, *P*< 0.001), and hospital length of stay (4 d, IQR 2–7, vs. 3 d, IQR 1–5, *P*=0.03), as shown in Table 2. There was also significant reduction in time to discontinuation or de-escalation of empiric antibiotic therapy (*P*=0.049) as shown in Figure 1.

Table 1. Baseline characteristics for patients with suspected bacterial meningitis

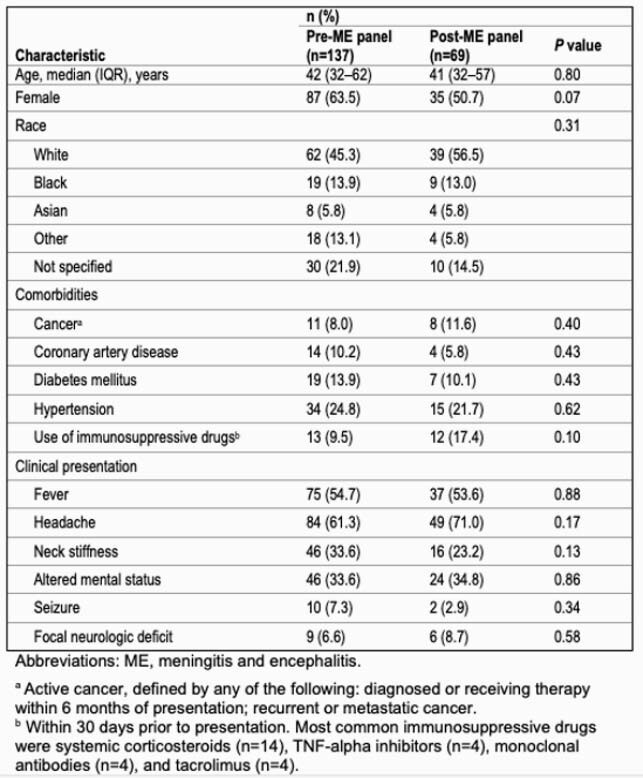

Table 2. Antimicrobial use and hospitalization outcomes

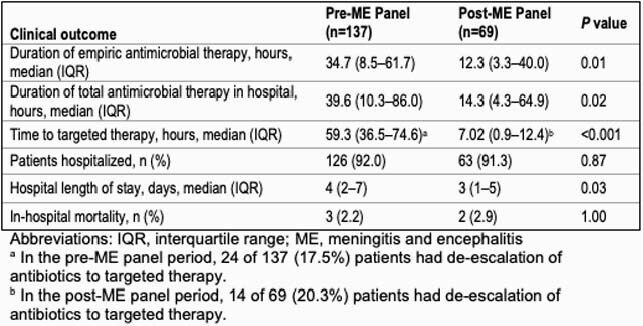

Compared to the pre-ME panel period, there were significant reductions in the post-ME panel period for the duration of empiric antibiotic therapy (P=0.01), time to targeted therapy (P<0.001), and hospital length of stay (P=0.03).

Figure 1. Probability of Empiric Antibiotic Therapy Between Pre- and Post-ME Panel Periods

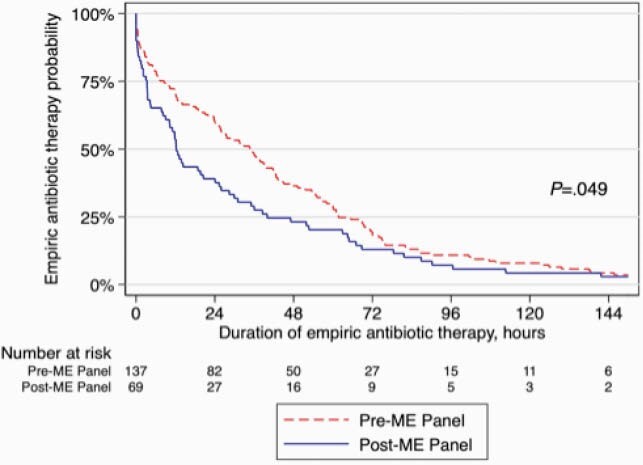

Kaplan-Meier analysis of the time from initiation of empiric antibiotic therapy to discontinuation or de-escalation of empiric antibiotic therapy between the pre- and post-ME panel periods. P value from log-rank test=0.049 (n=206). There was a significant difference in the time to discontinuation or de-escalation of empiric antibiotic therapy between the groups (sex- and immunosuppressant use-adjusted hazard ratio, 1.46 [95% confidence interval, 1.08–1.97]; P=0.01).

**Conclusion:**

The implementation of the FilmArray ME panel for suspected bacterial meningitis appears to reduce the duration of empiric antibiotic therapy, time to targeted therapy, and hospital length of stay compared to traditional culture-based microbiological testing methods.

**Disclosures:**

**Justin J. Choi, MD**, **Allergan** (Consultant, Grant/Research Support)**Roche** (Consultant, Grant/Research Support) **Lars Westblade, PhD**, **Accelerate Diagnostics Inc** (Grant/Research Support)**BioFire Diagnostics** (Grant/Research Support)**Hardy Diagnostics** (Grant/Research Support)**Roche** (Consultant, Advisor or Review Panel member)**Shionogi Inc** (Advisor or Review Panel member)**Talis Biomedical** (Advisor or Review Panel member) **Marshall J. Glesby, MD**, **Enzychem** (Consultant)**Gilead** (Grant/Research Support)**ReAlta Life Sciences** (Consultant)**Regeneron** (Consultant, Grant/Research Support)**Sobi** (Consultant)**Springer** (Other Financial or Material Support, Royalties)**UpToDate** (Other Financial or Material Support, Royalties)

